# Semantic Instance Segmentation of Kidney Cysts in MR Images: A Fully Automated 3D Approach Developed Through Active Learning

**DOI:** 10.1007/s10278-021-00452-3

**Published:** 2021-04-05

**Authors:** Adriana V. Gregory, Deema A. Anaam, Andrew J. Vercnocke, Marie E. Edwards, Vicente E. Torres, Peter C. Harris, Bradley J. Erickson, Timothy L. Kline

**Affiliations:** 1 Division of Nephrology and Hypertension, Mayo Clinic, Rochester, MN 55905 USA; 2 Department of Radiology, Mayo Clinic, Rochester, MN 55905 USA

**Keywords:** Autosomal dominant polycystic kidney disease, Magnetic resonance imaging, Three-dimensional instance segmentation, Cyst volume, Convolutional neural networks

## Abstract

Total kidney volume (TKV) is the main imaging biomarker used to monitor disease progression and to classify patients affected by autosomal dominant polycystic kidney disease (ADPKD) for clinical trials. However, patients with similar TKVs may have drastically different cystic presentations and phenotypes. In an effort to quantify these cystic differences, we developed the first 3D semantic instance cyst segmentation algorithm for kidneys in MR images. We have reformulated both the object detection/localization task and the instance-based segmentation task into a semantic segmentation task. This allowed us to solve this unique imaging problem efficiently, even for patients with thousands of cysts. To do this, a convolutional neural network (CNN) was trained to learn cyst edges and cyst cores. Images were converted from instance cyst segmentations to semantic edge-core segmentations by applying a 3D erosion morphology operator to up-sampled versions of the images. The reduced cysts were labeled as core; the eroded areas were dilated in 2D and labeled as edge. The network was trained on 30 MR images and validated on 10 MR images using a fourfold cross-validation procedure. The final ensemble model was tested on 20 MR images not seen during the initial training/validation. The results from the test set were compared to segmentations from two readers. The presented model achieved an averaged *R*^2^ value of 0.94 for cyst count, 1.00 for total cyst volume, 0.94 for cystic index, and an averaged Dice coefficient of 0.85. These results demonstrate the feasibility of performing cyst segmentations automatically in ADPKD patients.

**Supplementary Information:**

The online version contains supplementary material available at 10.1007/s10278-021-00452-3.

## Introduction

Autosomal dominant polycystic kidney disease (ADPKD) is a genetic disorder with approximately 140,000 people currently diagnosed in the USA [1]. It is characterized by the development of multiple cysts in the kidneys. As the number and size of the cysts increase, they interfere with the kidney’s ability to filter waste products from the blood and often leads to end-stage renal disease [2].

Many studies have shown that total kidney volume (TKV) is a useful prognostic biomarker in combination with age and estimated glomerular filtration rate for use in clinical trials and to predict renal function decline [3–5]. Although TKV is important, there is much more structural information that could be characterized. For instance, other image-derived biomarkers such as cyst number and size would provide further insight regarding the disease status [6]. ADPKD patients with similar TKVs may present with different kidney cyst compositions (i.e., few large cysts, many small cysts, or a combination of large and small cysts). This added information is likely clinically significant and currently is one factor used for the classification of typical and atypical imaging groups in ADPKD patients [7]. Moreover, Bae et al. [8] recently proposed that the exclusion of prominent exophytic cysts could improve the classification of patients. This cyst assessment, however, is often performed manually using the ellipsoid equation to approximate the cysts volumes or by user-dependent semi-automated methods.

During the past few years, machine learning (particularly deep learning) has been successfully implemented for the segmentation of natural images (i.e., images taken with an RGB camera) [9–11]. As a result, researchers in medical image analysis have translated and developed new technologies for the segmentation of several organs and tissues [12]. In ADPKD, fully automated methods using deep neural networks have been implemented to segment polycystic kidneys in CT scans with and without contrast enhancement reaching a Dice coefficient of 0.86 and 0.92, respectively [13, 14]. Fully automated models have also been implemented for T2-weighted MR images [15, 16] showing accurate results (Dice = 0.97).

Instance segmentation, however, goes one step further and is capable of segmenting examples of the same class (e.g., separating each instance of a car or cell in an image). Mask R-CNN [17] is a widely used method to generate instance segmentation by applying an object detection algorithm. Most applications of Mask R-CNN have been geared towards segmentation of natural images [18–21] and some for histopathology and 2D medical images [22–26]. For 3D images, however, the higher dimension of the scans can significantly increase GPU memory requirements. Only a couple of radiological studies have reported the use of Mask R-CNN, for the segmentation of lung nodules in chest CT [27] and hemorrhage evaluation in head CT [28]. The instance cyst segmentation task, on the other hand, poses a bigger challenge, not only because of the higher number of instances (up to thousands of cysts), which makes its implementation using an object detection approach unfeasible, but also due to the highly connected/clustered nature of the cysts in ADPKD.

Some studies have implemented more traditional segmentation approaches to generate instance and semantic cysts segmentations for ultrasound and CT imaging [29–32], by applying intensity- and shape-based methods. In MR imaging, two semi-automated approaches have been implemented to perform kidney cyst segmentations. The first approach used a k-means clustering method followed by connected components analysis to provide instance cyst segmentations [33]. The second approach used a region-based method to create binary signal-intensity maps to generate semantic cyst segmentations [34]. Most of these approaches rely on intensity information which may include other areas with similar intensity values such as the renal pelvis, and exclude some cysts with different signal intensities (e.g., hemorrhagic cysts). A fully automated method may be able to overcome these problems and provide more accurate cyst quantification parameters, particularly for severe cases where manual segmentation and semi-automated methods are not easily performed. Thus, in this paper, we propose a deep learning model for 3D instance cyst segmentation in order to measure total cyst volume (TCV) as well as cyst count and cystic index.

## Materials and Methods

This retrospective imaging study was reviewed and approved by our Institutional Review Board. 3D manual segmentation of individual cysts in MR images from ADPKD patients is a very difficult and time consuming task. To alleviate the process of creating a reference standard dataset an initial model was implemented. The initial model was trained on 15 3D MR images, validated on 5 images and tested on an additional 5 images. The output and framework of the initial model were then used for the improved final model.

### Initial Model: MRI Dataset

Twenty-five (*n* = 25) patients with ADPKD and available MRI imaging representative of different stages of cyst development and kidney enlargement were identified from our PKD Imaging Database. Only coronal T2-weighted fat saturated scans showing no blur artifacts and acquired with a 3-T field strength (*n* = 23) and 1.5-T field strength (*n* = 2) were selected for this study. As images were taken over a period of several years, the specific MR acquisition varied, but all consisted of matrix size 256 × 256 × Z (with FOV and Z large enough to cover the full extent of the kidneys). The median image voxel size was 1.37 mm (range 0.82 to 1.56 mm) in-plane, with a median slice thickness of 3.0 mm (range 3.0 to 9.0 mm).

**Fig. 1:**
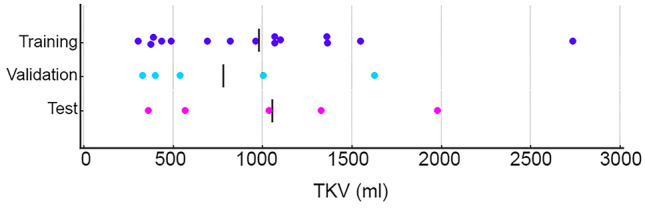
Visualization of the TKV distributions for training, validation, and test datasets for the initial model development. The bars represent the mean TKV in each set

TKVs obtained by applying automatic kidney segmentation [35] were available for all cases. The scans were sorted based on their TKV and randomized into training (*n* = 15), validation (*n* = 5), and test (*n* = 5) sets as shown in Fig. 1 by stratified sampling to ensure similar kidney size distribution among sets.

### Initial Model: Reference Standard

#### Cyst Segmentation: Test Set

The cysts were manually traced by two trained readers using the software PKD-GUI (available online at https://github.com/TLKline/InstanceCystSeg) developed in-house. The software allows for freehand tracing, and the final segmentations were saved as compressed NIFTI files. The readers used only one label to trace all cysts excluding renal pelvis, vessels, normal renal tissue, and high intensity areas smaller than 4 voxels which could not be differentiated from image noise. Additionally, the readers were blinded to patient information and the other reader’s tracings.

#### Instance Cyst Segmentation: Test Set

An initial instance cyst segmentation was generated from the manual cyst tracings by applying a 3D watershed algorithm to a 3D Euclidean distance map based on the MRI and cyst segmentation images (MATLAB, v.R2018b). The algorithm assigned different labels to each cyst in ascending order. The instance cyst segmentation was later reviewed by the same reader that performed the manual cyst segmentation and was corrected when necessary (i.e., by splitting or merging labels) using the in-house software described previously.

### Initial Model: Data Pre-processing

#### Reference Standard: Training and Validation Sets

To expedite the cyst segmentation process for the training and validation sets, a previously developed automated semantic cyst segmentation algorithm was used to perform the one label cyst segmentation [36]. Next, the resulting segmentation underwent the 3D watershed process described in the previous section. Finally, the images were reviewed by Reader 1.

Prior to training the deep learning model, the segmentation masks were converted to semantic segmentations as cyst-edge and cyst-core. The edge was obtained by subtracting the cyst mask with the 3D dilated cyst mask (using a 6-connected structuring element). The cyst core was the same as the cyst mask. The conversion process was performed in a cyst-by-cyst fashion (MATLAB, v.R2018b).

### Initial Model: Semantic Instance Cyst Segmentation

#### Deep Learning Model

**Fig. 2:**
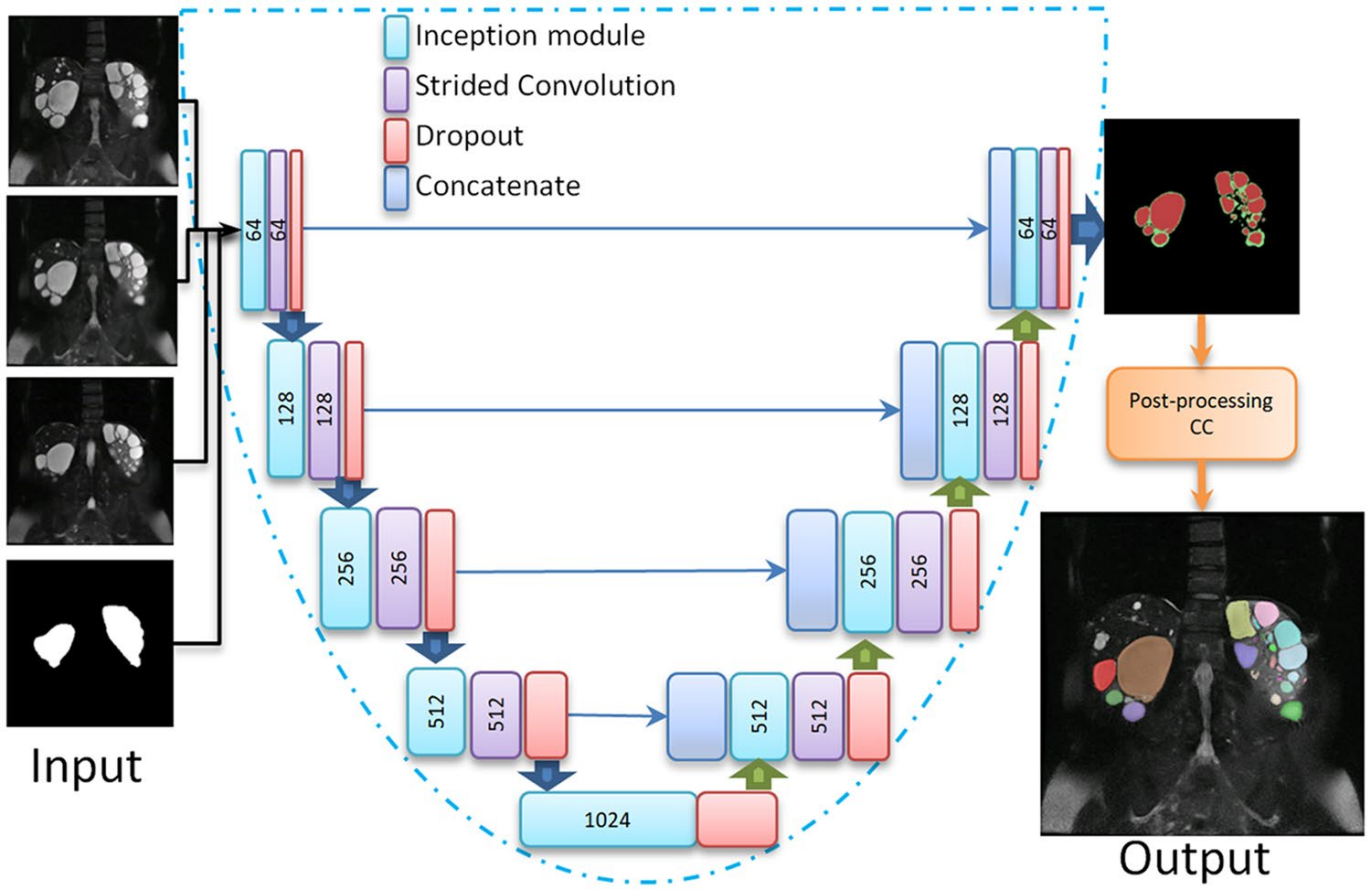
Architecture of the inception U-Net. The input is a 4-channel structure that consists of the MRI slice to be segmented, the corresponding kidney segmentation mask and the posterior and anterior MRI slices. The network includes inception modules followed by strided convolutions and dropout. The output of the U-Net is the edge-core segmentation. The output of the entire algorithm is the 3D instance cyst segmentation

The deep learning model is based on the U-Net neural network [37] using inception modules similar to the previous work [36]. The input to the model consists of 4 images: 3 consecutive MR slices and 1 kidney segmentation mask corresponding to the middle MR input image. The output of the model is the predicted edge-core image for the middle input image (Fig. 2). For the first and last MRI slices of an exam, the first and third input images were zero-padded matrices, respectively.

#### Training the Model

The model was trained on an NVidia graphical processing unit (model: Tesla P40) using the Keras python library and Python 3.6.1. The number of epochs was set to 200 with a batch size of 6. The evaluation of the segmentation was assessed by the Jaccard loss function,

$$Jaccard\;loss=\frac{\sum_i^Np_ir_i}{\sum_i^Np_i+\sum_i^Nr_i-\sum_i^Np_ir_i}$$

where *r* is the reference segmentation, *p* is the predicted segmentation, and *N* is the number of voxels. The Adam optimizer was used with learning rate = 1e−3.

### Initial Model: Data Post-processing

In order to assign an individual label to each cyst, a connected components algorithm with 6 neighbor voxel connectivity was applied to the cores segmentation at the output of the deep learning model.

### Final Model: MRI Dataset

Thirty five (*n* = 35) 3D MR images from patients with ADPKD and available 1.5-T or 3-T T2-weighted coronal fat saturated images representative of a wider range of different stages of cyst development and kidney enlargement were added to the data set from the initial model. TKV segmentations were available for all cases. In total, 60 MR images with matrix size of 256 × 256 × Z or 512 × 512 × Z (with FOV and Z large enough to cover the full extent of the kidneys) comprised the final dataset. The median image voxel size was 1.41 mm (range 0.8 to 1.88 mm) in-plane, with a median slice thickness of 4.0 mm (range 3.0 to 9.0 mm).

**Fig. 3:**
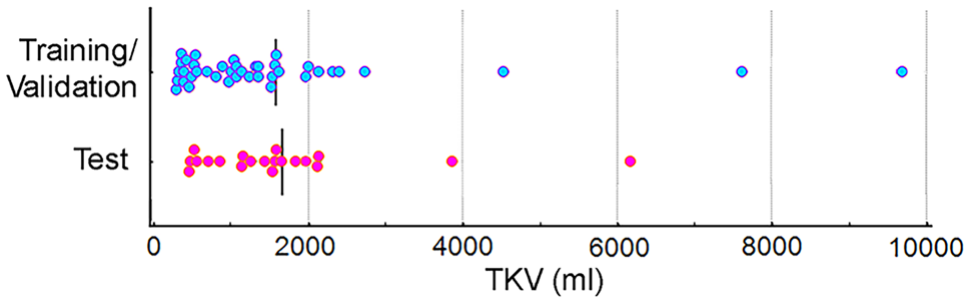
Visualization of the TKV distributions for training/validation (blue dots) and test (pink dots) for the final model data set. The bars represent the mean TKV in each set

The images were sorted based on their TKV measurements. Forty MR images (including the 25 images from the initial model) were used for training and validation using a fourfold cross-validation technique. The remaining 20 MR images were used for testing. Figure 3 shows the TKV distributions for all the sets.

### Final Model: Reference Standard

Instance cyst segmentations of the newly added 35 MR images were generated using the initial model. Two independent readers revised the testing images (*n* = 20) and one reader revised the training/validations images (*n* = 15). Finally, all 60 instance cyst segmentation masks and corresponding MR images were up-sampled to a 512 × 512 × 3Z matrix size by using bilinear and bicubic interpolations, respectively. An opening operation was applied to each cyst instance to preserve the exact instance segmentation after post-processing (i.e., dilation after erosion of the core).

The instance segmentations were converted to semantic edge-core segmentations where the core was the result of the 3D eroded cyst instances and the edge was obtained by adding the 3D inner and 2D outer cyst instance edges. The 3D and 2D morphological operations were performed using a 3 × 3 × 3 voxel 6-connectivity kernel and a 3 × 3 voxel 4-connectivity kernel, respectively.

### Final Model: Ensemble Learning

**Fig. 4:**
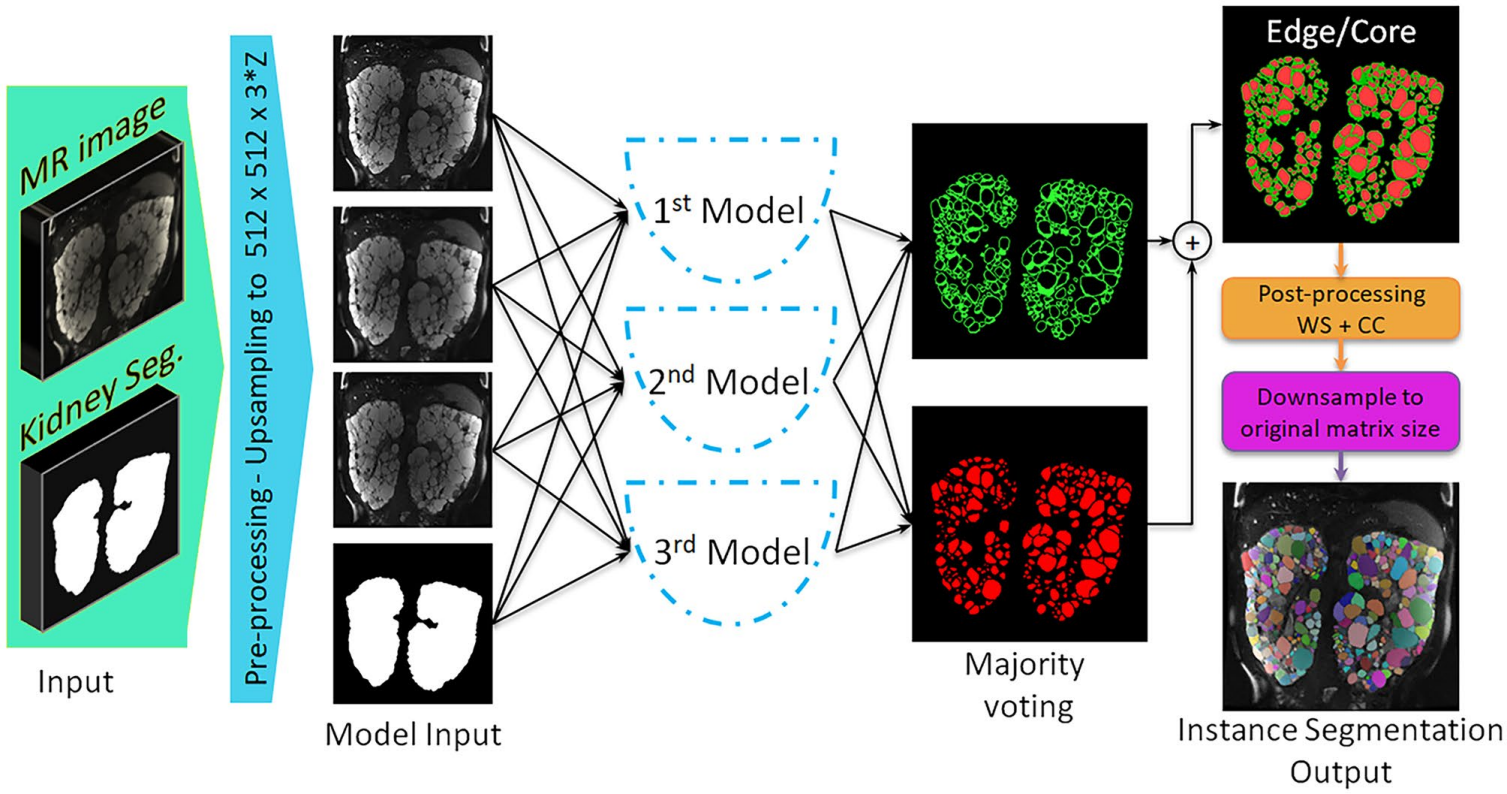
Architecture of the ensemble model. The MR image and the corresponding kidney segmentation are up-sampled by bilinear and bicubic interpolation, respectively, to two matrices of size 512 × 512 × 3Z. The input to the ensemble model is three consecutive MR slices and the kidney mask corresponding to the second input MR slice. Three inception U-Nets with architecture shown in Fig. 2 generate 3 edge-core prediction masks for the second input MR slice. The final prediction is the outcome of a majority voting algorithm at the voxel level. A 3D Watershed algorithm followed by a 3D connected components analysis is performed on the core prediction to obtain the instance cyst segmentation mask; then each instance is dilated in 3D by one voxel to incorporate the edges in the final instance cyst segmentation. Lastly, the predicted final instance segmentation is downsampled to the original MR volume

Four models were obtained from the fourfold cross-validation sets using the U-Net architecture shown in Fig. 2. The three models with the best performance on the validation set were combined as the final ensemble model. The output was generated by majority voting of the three edge and core segmentations. A diagram of the final ensemble model is shown in Fig. 4. The ensemble model is available online, located at https://github.com/TLKline/InstanceCystSeg.

### Final Model: Post-processing

From the initial model results, it was observed that, due to the lower image resolution on the Z axis, some cyst cores were connected to posterior/anterior cysts. In order to improve the conversion from semantic to instance segmentation and with the knowledge that cysts have a shape similar to a sphere, we first calculated the 3D Euclidean distance map on the cores segmentation. Then, the 3D Watershed algorithm was applied to split any cysts that might not be separated by the edge segmentation. Next, with the objective to preserve cyst cores mainly composed by a few voxels that might be lost after the Watershed algorithm, a connected components algorithm with 6-nearest neighbor connectivity was applied to the difference between the predicted core map and the 3D Watershed output. The labels of the 3D connected components instance segmentation were assigned starting from one plus the last label from the 3D Watershed output. Then, the connected components instance segmentation was added to the watershed instance segmentation. Finally, all cyst instances were dilated in 3D by a 3 × 3 × 3 voxel 6-connected structuring element. The last post-processing step was the downsample of the image to its original shape.

### Statistical Analysis

The initial and ensemble model were evaluated by comparing the automated instance cyst segmentations to the 3D reference standard cyst segmentations. The instance segmentations were binarized for voxel-wise comparison using the Jaccard index, Sorensen-Dice coefficient, precision, recall, and absolute relative volume change. TCVs, calculated as the sum of all segmented voxels multiplied by the voxel volume, were evaluated by Bland–Altman analysis and linear regression. The total number of cysts was evaluated using linear regression. Lastly, the cystic index, defined as the percent of cyst volume over total kidney volume, was evaluated by a Bland–Altman analysis and linear regression. The statistical analysis was performed using JMP (JMP, version 14) and MedCalc (MedCalc Statistical Software, version 19.1.3).

## Results

### Initial Model: 5-Case Test Set—Cyst Segmentation

The results of the TCVs calculated from the segmentations performed by Reader 1, Reader 2, and those automatically obtained with the initial mode (ordered based on ascending TKV) are observed in Table 1. Bland–Altman analysis resulted in a bias and precision of − 8.9% ± 7.0% between Reader 1 and Reader 2, 0.9% ± 32.2% between Reader 1 and the initial model, and 9.5% ± 30.8% between Reader 2 and the initial model.

**Table 1:** TCV measurements from Reader 1, Reader 2, and the initial model

Test set cases	Reader 1 (mL)	Reader 2 (mL)	Initial model (mL)
K1	43.1	50.7	74.4
K2	105.8	124.2	88.1
K3	614.7	635.4	603.2
K4	726.6	793.8	530.2
K5	1723.1	1732.5	1622.6

Table 2 summarizes the comparison of similarity metrics between Reader 1, Reader 2, and the initial model for the 5-case test set. Similar performance is observed between the readers and between each reader and the initial model.

**Table 2:** Similarity analysis between the reference standard and the initial model

Similarity metric (mean ± SD)	Reader 1 vs Reader 2	Reader 1 vs initial model	Reader 2 vs initial model
Dice	0.87 ± 0.05	0.84 ± 0.09	0.82 ± 0.09
Jaccard	0.77 ± 0.09	0.74 ± 0.14	0.71 ± 0.14
Precision	0.84 ± 0.08	0.87 ± 0.16	0.88 ± 0.14
Recall	0.91 ± 0.03	0.85 ± 0.11	0.80 ± 0.12
ARVC	0.09 ± 0.07	0.25 ± 0.15	0.24 ± 0.16

*ARVC* absolute relative volume change

### Initial Model: 5-Case Test Set—Cyst Count Analysis

The total number of cysts were K1, 102, 115, 224; K2, 96, 99, 105; K3, 274, 178, 183; K4, 676, 755, 721; and K5, 279, 177, 145 cysts, for Readers 1, 2, and the initial model, respectively. Linear regression analysis resulted in *R*^2^ = 0.94 between Reader 1 and Reader 2, *R*^2^ = 0.82 between Reader 1 and the initial model, and *R*^2^ = 0.96 between Reader 2 and the initial model.

**Fig. 5:**
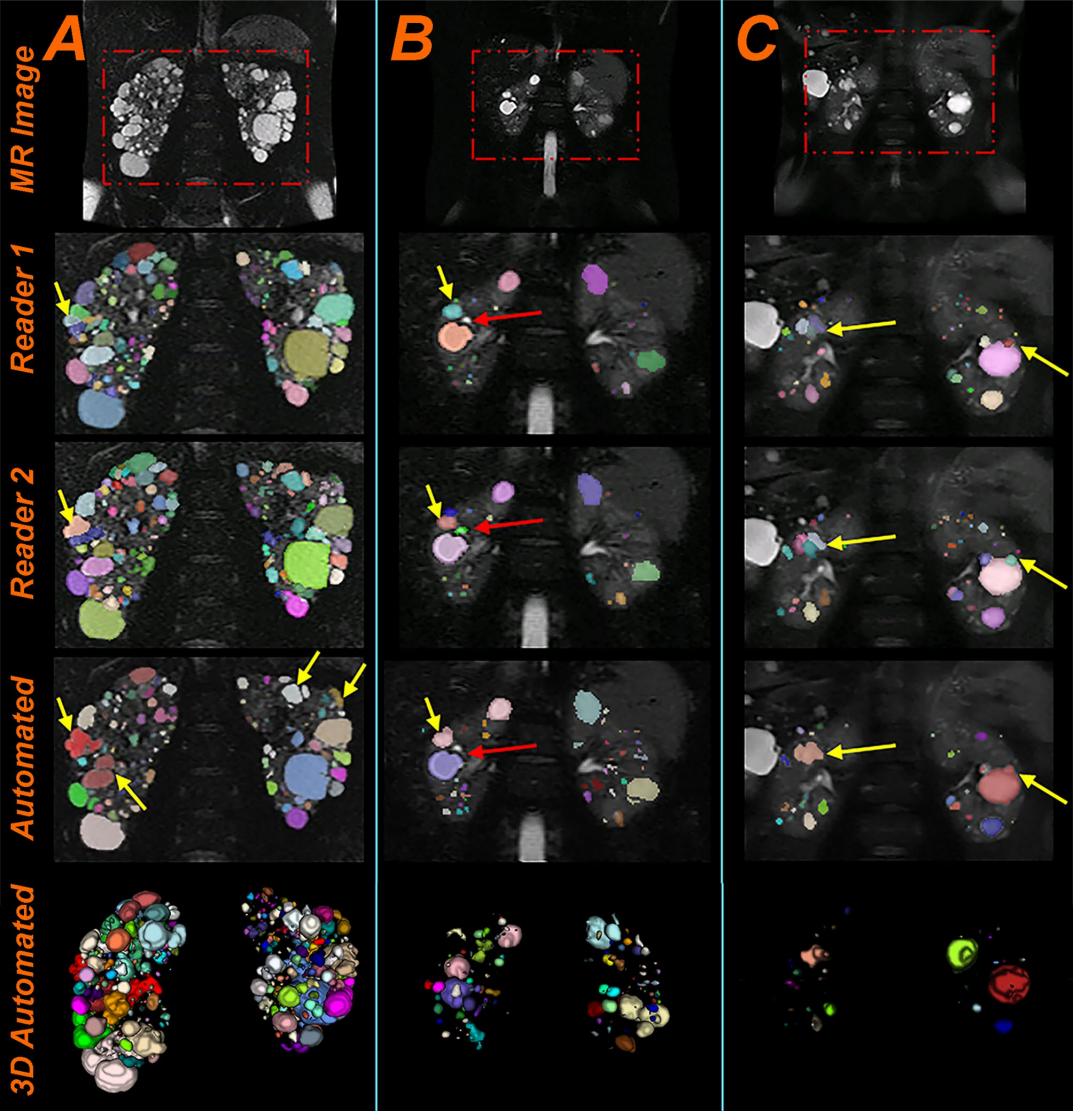
Three **e**xample images from the test set showing the segmentations differences between Reader 1, Reader 2, and the initial automated model. The cyst colors show the difference between adjacent cysts and are assigned randomly; thus, we do not expect them to be the same between the readers and the automation. The arrows point to some differences between the segmentations. **a** Example of a severe ADPKD case. The average Dice coefficient from the 3 segmentations was 0.82. **b** Example of a mild PKD case. Average Dice coefficient was 0.89. **c** Example of a mild PKD case. Average Dice coefficient was 0.74

A representative slice from three testing cases showing differences between Reader 1, Reader 2 and the initial model is presented in Fig. 5. The yellow arrows point to cysts that were segmented differently between readers or between the readers and the automated method. In most cases, the model assigned one label to clustered cysts. The red arrow shows a difference between Reader 1 and Reader 2, where Reader 1 excluded the bright intensity area seen as part of the renal pelvis but Reader 2 interpreted the area as a cyst.

### Final Model: 20-Case Test Set—Cyst Segmentation

Table 3 summarizes the similarity metrics between Reader 1, Reader 2, and the final model for the 20-case test set. We observed that the similarity metrics between Reader 1 and Reader 2 show higher agreement compared to the results from the initial 5-case test set. This could be caused by a bias effect since both readers started from an initial instance segmentation generated by the initial model. Furthermore, we observed similar performance between the final model and both readers, taking into consideration that the test set includes a wide range of ADPKD phenotypes.

**Table 3:** Similarity analysis between Reader 1, Reader 2, and the ensemble model

Similarity metric (mean ± SD)	Reader 1 vs Reader 2	Reader 1 vs final model	Reader 2 vs final model
Dice	0.91 ± 0.06	0.84 ± 0.09	0.85 ± 0.07
Jaccard	0.84 ± 0.1	0.73 ± 0.12	0.74 ± 0.1
Precision	0.97 ± 0.03	0.88 ± 0.07	0.84 ± 0.08
Recall	0.86 ± 0.09	0.80 ± 0.11	0.86 ± 0.08
ARVC	0.11 ± 0.08	0.12 ± 0.7	0.07 ± 0.09

*ARVC* absolute relative volume change

**Fig. 6:**
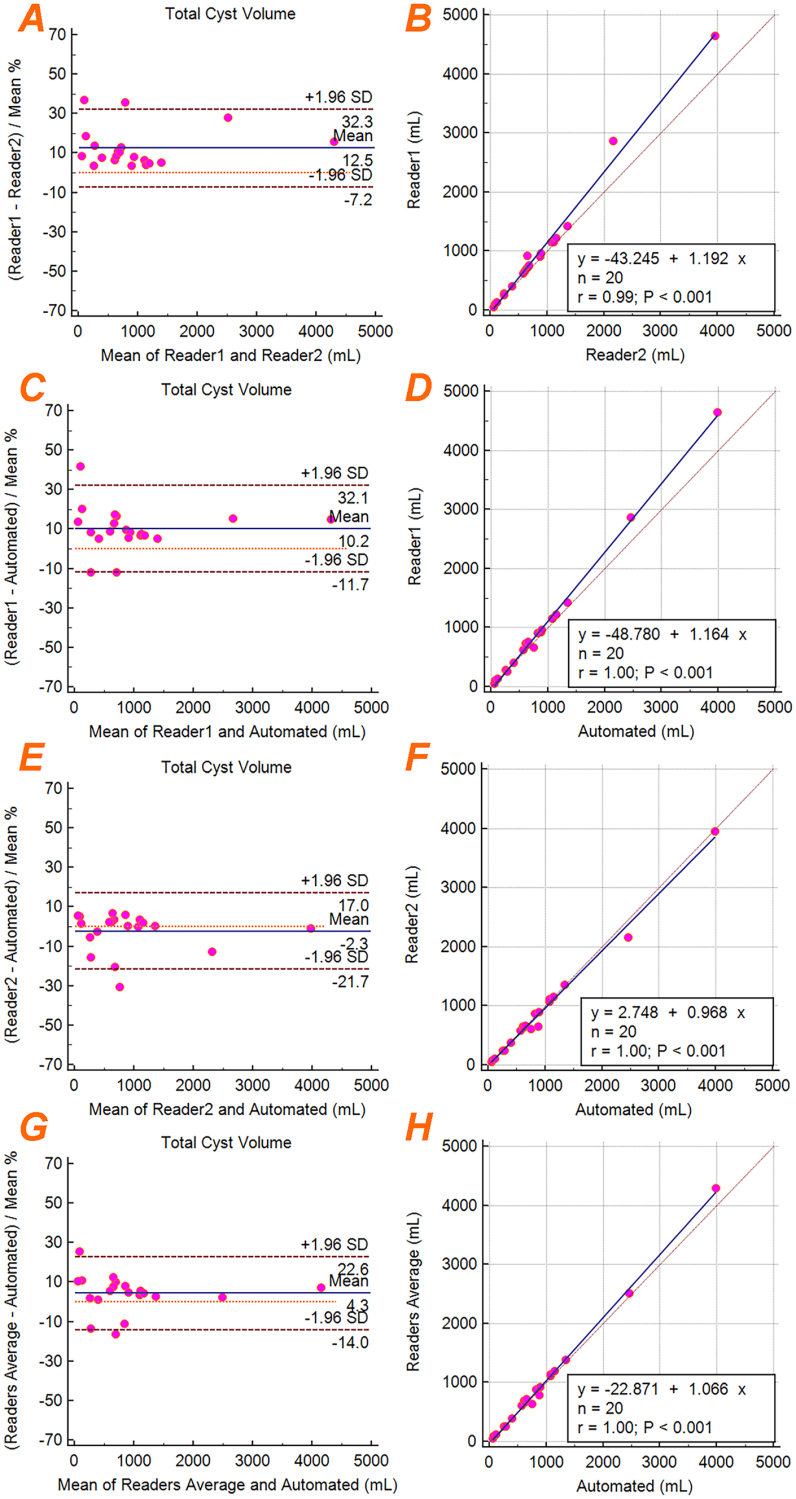
Bland–Altman plots (**a**, **c**, **e**, **g**) showing the TCV agreement between Reader 1, Reader 2, and the final automated ensemble model. Linear regression plots (**b**, **d**, **f**, **h**) show good TCV correlations between Reader 1, Reader 2 and the automated ensemble model

Bland–Altman analysis of the TCV resulted in a bias and precision of 12.5% ± 10.1% between Reader 1 and Reader 2, 10.2% ± 11.2% between Reader 1 and the ensemble model, − 2.3% ± 9.9% between Reader 2 and the ensemble model, and 4.3% ± 9.3% between the average of Reader 1 with Reader 2 and the ensemble model. Linear regression analysis resulted in perfect agreement (*R*^2^ = 1.00) between Reader 1 and the ensemble model, Reader 2 and the ensemble model, and the readers average with the ensemble model. Regression analysis between Reader 1 and Reader 2 showed almost perfect correlation with *R*^2^ = 0.98. Figure 6 shows the Bland–Altman and linear regression plots for TCV.

**Fig. 7:**
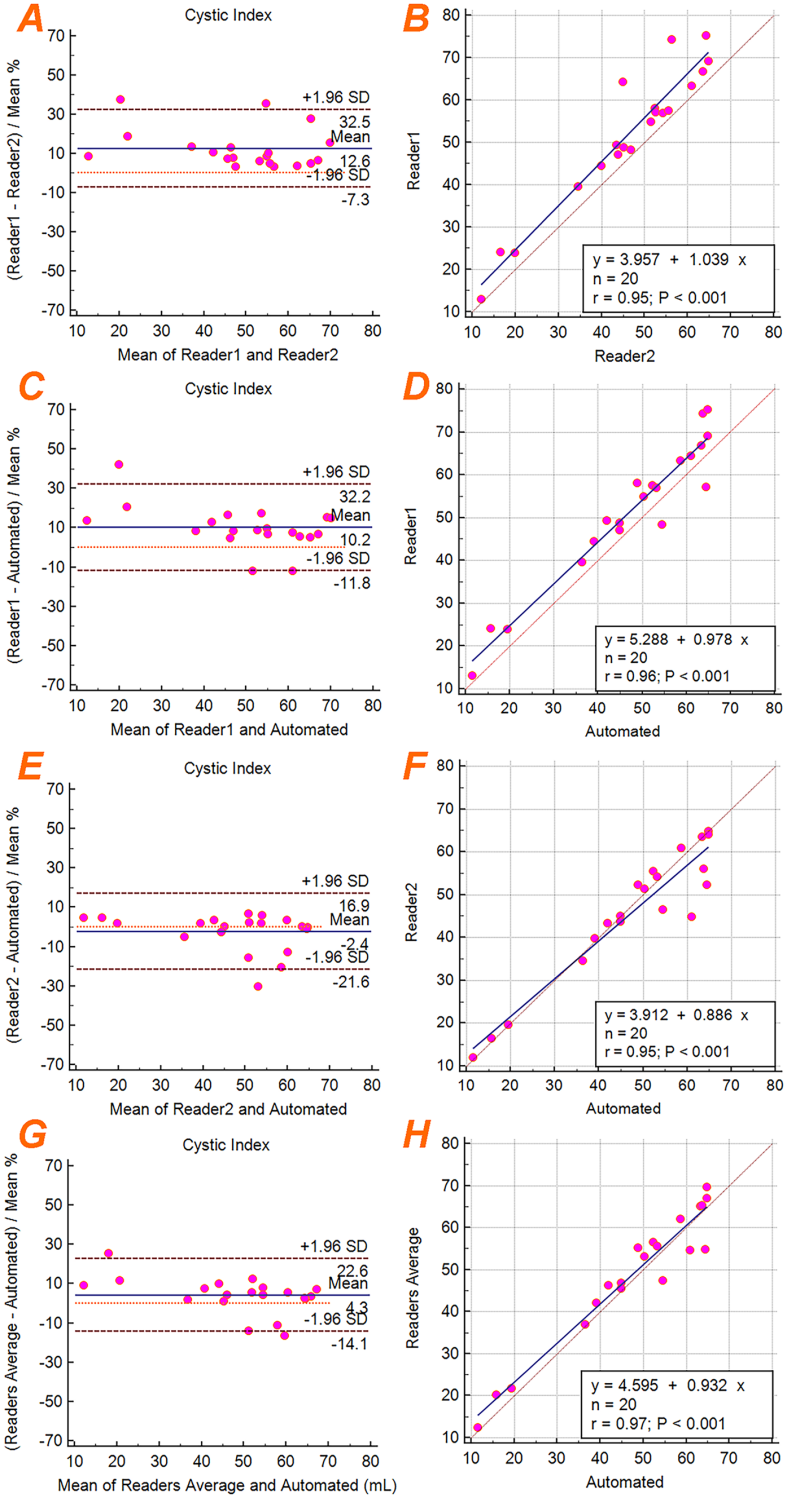
Bland–Altman plots (**a**, **c**, **e**, **g**) showing the cystic index agreement between Reader 1, Reader 2, and the final automated ensemble model. Linear regression plots (**b**, **d**, **f**, **h**) showing the cystic index correlations between Reader 1, Reader 2, and the automated ensemble model

Bland–Altman analysis of the cystic index resulted in a bias and precision of 12.6% ± 10.5% between Reader 1 and Reader 2, 10.2% ± 11.2% between Reader 1 and the ensemble model, − 2.4% ± 9.8% between Reader 2 and the ensemble model, and 4.3% ± 9.4% between the both readers average and the ensemble model. The best cyst index correlation was between the average of both readers and the ensemble model (*R*^2^ = 0.94). Linear regression analysis resulted in *R*^2^ = 0.90 between Reader 1 and Reader 2, *R*^2^ = 0.92 between Reader 1 and the ensemble model, *R*^2^ = 0.90 between Reader 2 and the ensemble model. Figure 7 shows the Bland–Altman and linear regression plots for cystic index.

### Final Model: 20-Case Test Set—Cyst Count Analysis

**Fig. 8:**
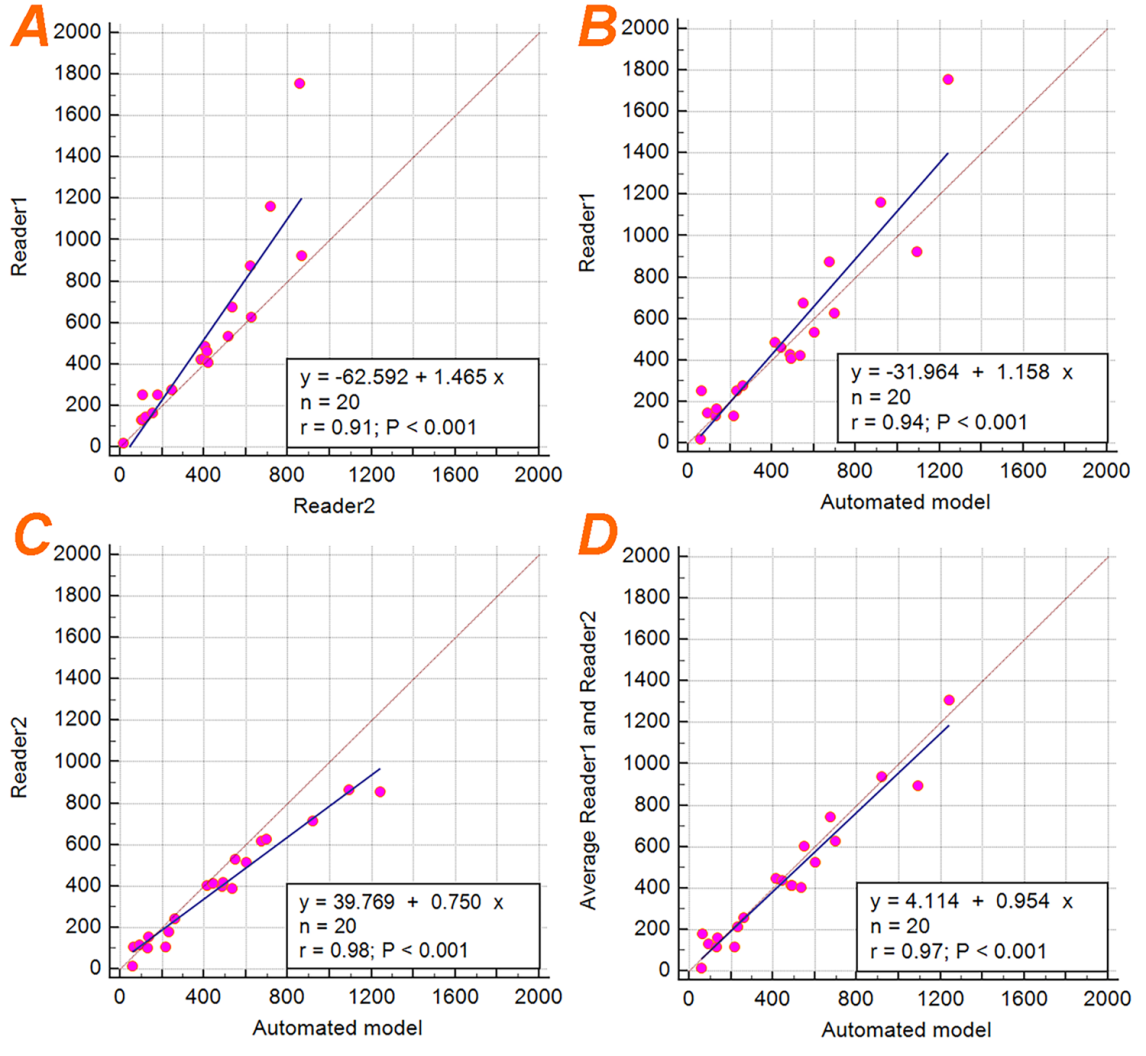
Linear regression plots showing the cyst count correlations between Reader 1 and Reader 2 (**a**), Reader 1 and the ensemble model (**b**), Reader 2 and the ensemble model (**c**), and the average count between Reader 1 and Reader 2 compared to the ensemble model (**d**)

The total number of cysts obtained from the instance cyst segmentations were analyzed by correlation (Fig. 8). Reader 2 and the ensemble model showed the strongest correlation with an *R*^2^ = 0.96, where the model detected slightly more cysts than Reader 2. Comparison between Reader 1 and the final ensemble model show good agreement (*R*^2^ = 0.88), where Reader 2 identified more cysts than the ensemble model for most cases. The inter-observer average cyst count, however, showed excellent agreement with the ensemble model cyst count (*R*^2^ = 0.94) (Fig. 8d). Lastly, the comparison between Reader 1 and Reader 2 showed the lowest agreement with an *R*^2^ = 0.83, where Reader 1 identified more cysts than Reader 2.

**Fig. 9:**
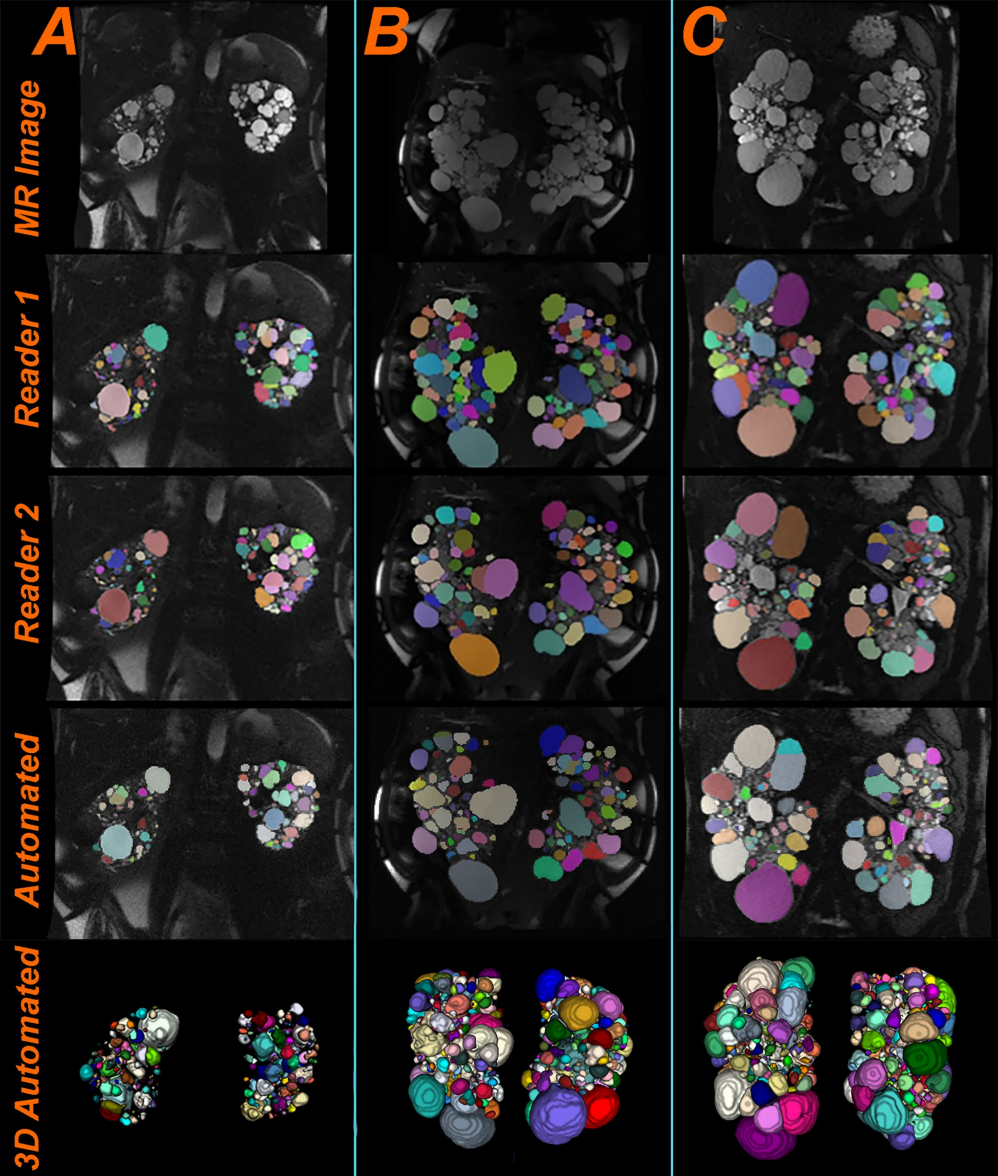
Test set example images showing three instance segmentations generated by Reader 1, Reader 2, and the ensemble automated model. The cyst colors are unique to each cyst and are assigned randomly; thus, we do not expect them to be the same between readers and the automation. **a** Example of a moderate ADPKD case. The average Dice coefficient from the 3 segmentations was 0.88. **b** Example of a severe ADPKD case. Average Dice coefficient was 0.87. **c** Example of a severe ADPKD case. Average Dice coefficient was 0.87

A representative slice from three testing cases is presented in Fig. 9. We can observe that the automated method provides good instance segmentations for ADPKD cases with multiple-clustered cysts (which are the most challenging cases for the algorithm) and the results look visually on par with the instance segmentations from the two readers.

**Fig. 10:**
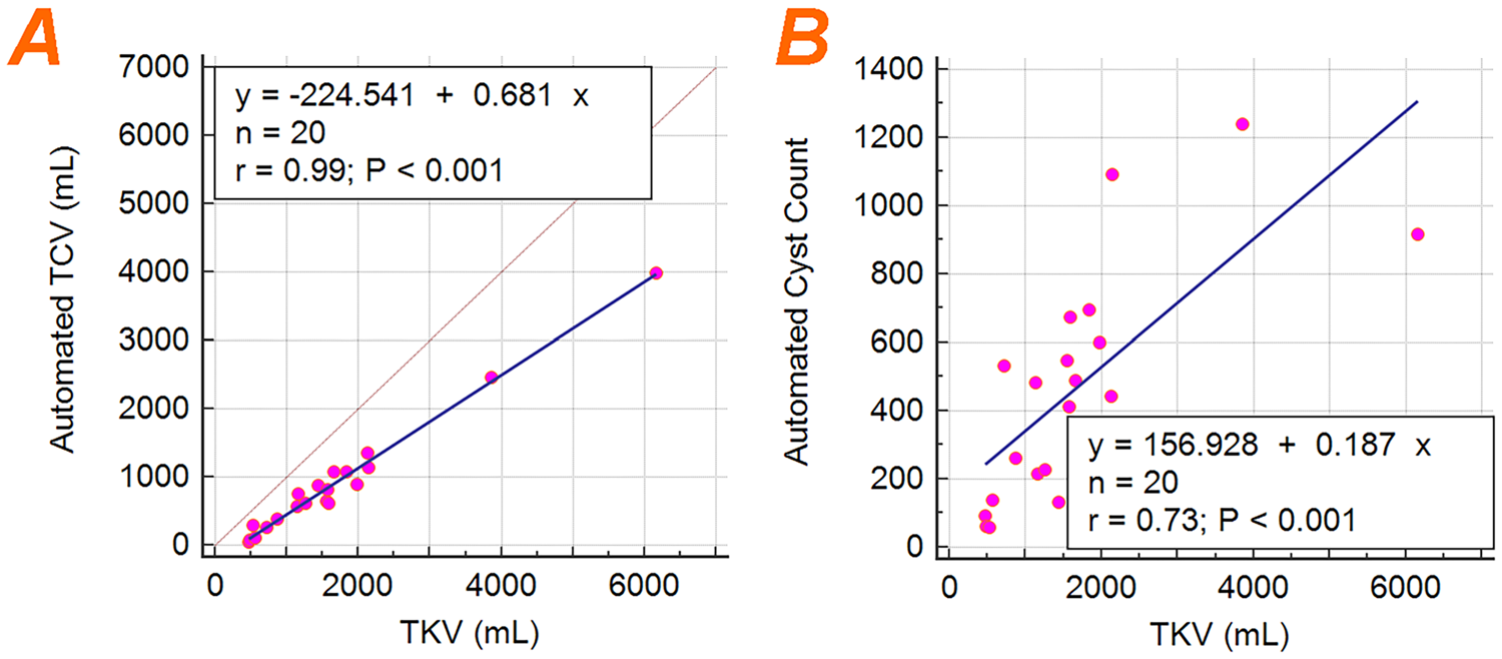
Linear regression plots between **a** TKV and the ensemble model TCV and **b** TKV and ensemble model cyst count

Finally, a comparison between TKV (i.e., the main imaging biomarker for ADPKD assessment) and TCV and cyst count is shown in Fig. 10. Linear regression analysis shows that 98% of the variation in TCV is explained by its linear association with TKV; however, only 53% of the variation in cyst count is explained by its linear association with TKV. This provides evidence that cyst count can offer additional phenotypic information beyond TKV.

## Discussion

In this study, we present the first deep learning approach to segment multiple clustered cyst instances in MR images for ADPKD patients. The comparison of the predicted TCV, cyst count, and cystic index to the results from two readers showed that an automated approach can produce accurate 3D instance cyst segmentations.

Deep learning models require large amounts of data to produce the best results. The lack of 3D annotated images was a major bottle neck at the beginning of the study. The 5 manually segmented images in the initial test set required several weeks to complete and were very labor intensive. The most challenging images are the ones from advanced/severe ADPKD patients where multiple cysts are clustered together and the boundaries are not as defined as when the cysts are surrounded by kidney parenchyma. To overcome this obstacle, the study was divided in two sections to solve this problem by active learning. The first part of the study included an initial model trained on a small dataset. The second part of the study included the development of the final model using a larger dataset, where the reference standard was initialized by the initial model.

The results from this study are on par with the results obtained by Bae et al. [33], where he proposed a semi-automated method to generate instance cyst segmentations. The TCV bias reported using the semi-automated segmentation compared to a region-based volume was − 9% with an *R*^2^ = 0.98 for cyst count. In our study, the TCV bias between the readers and the fully automated method were 10.2% and − 2.3%, respectively, with an *R*^2^ = 0.94 for cyst count. It is worth mentioning that the testing set in our study included cases with higher TCV and larger number of cysts, and the proposed ensemble model does not require any user input.

**Fig. 11:**
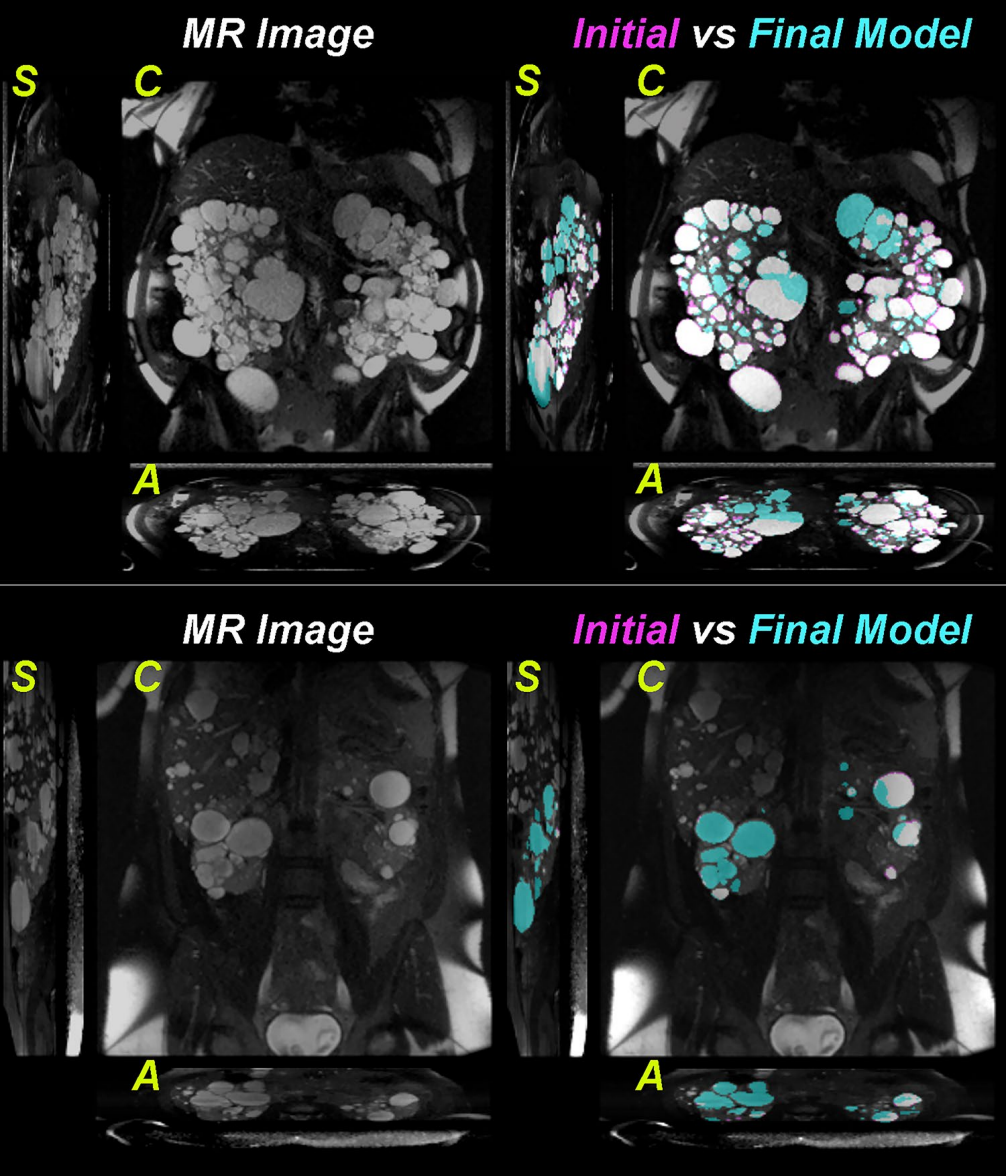
Two example images acquired with 1.5-T MR scanners. In order to show the cyst segmentation difference between the initial model and final model, the instance cyst segmentations were converted to binary maps. The pink color shows the initial model prediction and the teal color shows the final model prediction. The agreement between the two models is shown as white. S sagittal view, C coronal view, A axial view

Cysts in MR images can be shown with different intensities depending on the cyst composition. Most cysts in ADPKD are simple cysts (i.e., do not contain solid components) and are shown as hyperintense regions on T2-weighted MR images. Complicated cysts (e.g., proteinaceous, hemorrhagic, infected cysts), however, are often seen as hypointense regions [38]. Another source of difference in cyst intensity is observed depending on the strength of the magnetic field of the MR scanner. Three-tesla scanners have a higher magnetic field strength and provide higher SNR, thus better image quality and cyst contrast. To provide accurate cyst segmentations, the automated method is required to be intensity invariant. With the goal of increasing model generalizability, the final ensemble model was trained on images acquired with 1.5-T and 3-T MR scanners. Moreover, we included images presenting complicated cysts in a wide range of ADPKD phenotypes and images with different resolutions. Furthermore, the cohort included cases with TKVs ranging from volumes close to that of normal kidneys (~ 290 mL) up to cases reaching almost 10,000 mL, which practically covers the whole range of TKVs in ADPKD. Two examples of the improved generalizability of the ensemble model over the initial model can be observed in Fig. 11, where the final model was able to identify cysts with lower intensities.

**Fig. 12:**
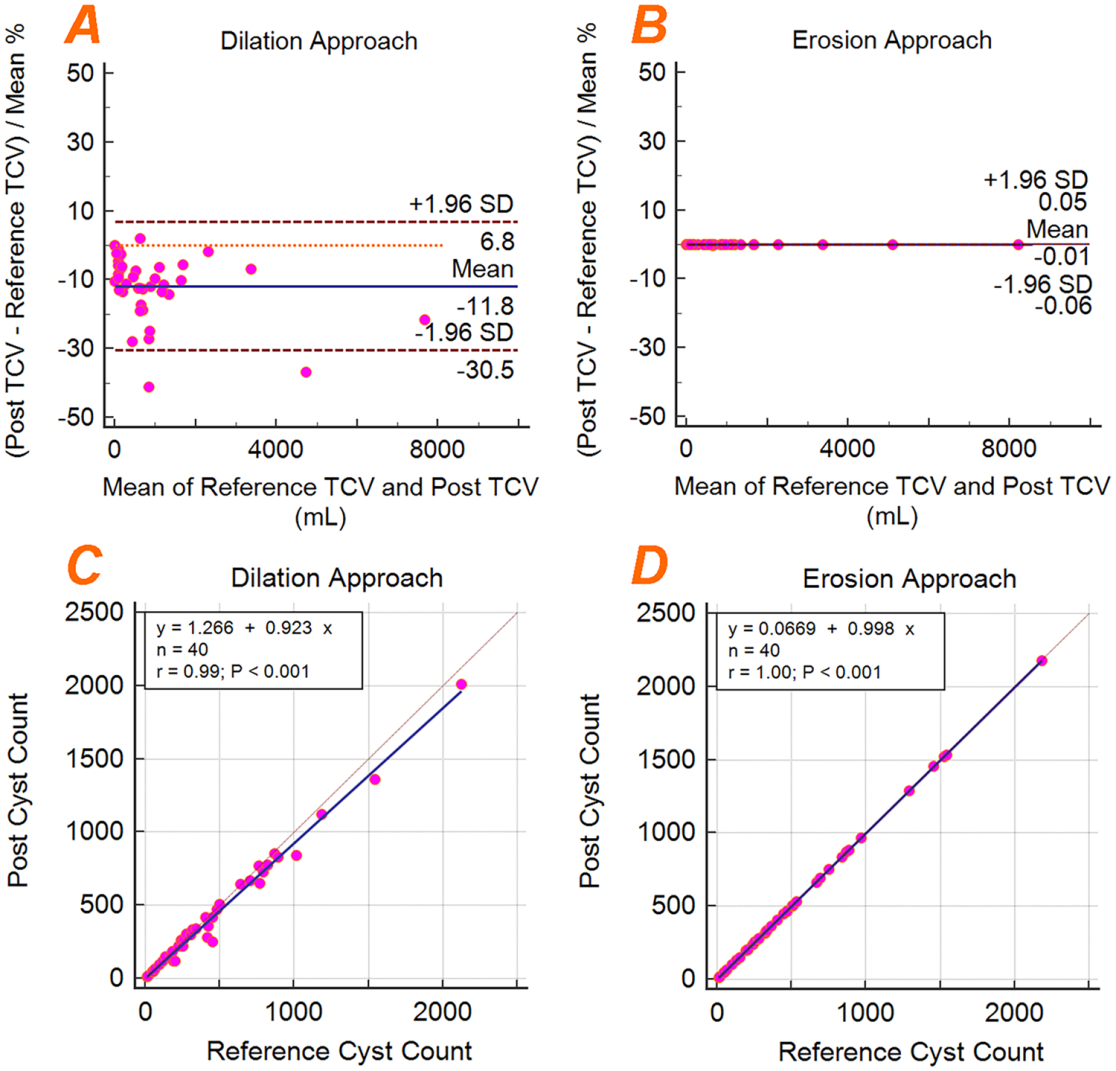
Effect of applying the semantic segmentation conversion to 40 reference standard instance cyst segmentations (training and validation sets) and back-converting to the instance segmentation (“Post”). **a** The dilation morphology approach resulted in a TCV bias and precision of − 11.8% ± 9.5%. **b** The erosion morphology approach resulted in a TCV bias and precision of − 0.01% ± 0.02%. **c**, **d** Linear regression showing the cyst count before and after applying the dilation and erosion morphology approaches, respectively

The conversion from instance cyst segmentation to semantic edge-core segmentation for training the model is another key step, particularly for clustered cysts. The semantic edge segmentation for the initial model was generated by applying a dilation morphology operator. This approach was chosen because some cysts are present on only one slice and an erosion approach would not preserve these cysts after the conversion. The dilation method provided a good estimation of the cyst edges on the in-plane view (*x* and *y* view) but, due to the lower resolution on the Z dimension, the posterior and anterior cyst edges interfered with adjacent cysts. For the final model, we decided to generate the semantic edge segmentation applying the erosion morphology operator. To preserve all cysts, the MR images and the corresponding instance segmentations were interpolated to a size 512 × 512 × 3Z. Thus, cysts present on only one slice would be interpolated to 2 additional slices. We up-sampled the images with in-plane matrix size of 256 × 256 to 512 × 512 because the ensemble model requires a consistent input matrix size. To account for the higher class imbalance (core class >> edge class) due to the interpolation, we performed a 2D dilation of the edges. The improved preservation of TCV and cyst count of the erosion approach over the dilation approach can be observed in Fig. 12.

The design of the final model included the voting ensemble of three different models. These models were the top three best performing models on the validation set obtained from the fourfold cross-validation method. The ensemble model with the majority voting algorithm were implemented since voting ensembles are characterized by being more stable and have better performance than single models [36]. The agreement results between the individual models, the ensemble model and the readers can be found in the supplemental material section.

One limitation of MR imaging is the low resolution to show microcysts. The mean voxel volume in this study was 0.0076 ± 0.0034 mL. However, due to the inherent noise present on MR images (i.e., salt and pepper noise), we implemented a lower cyst size threshold of 4 voxels (mean in-plane diameter 2.76 ± 0.48 mm) after post-processing.

Object detection approaches to perform instance segmentations for overlapping and clustered instances are becoming increasingly popular due to their high performance on natural and 2D medical images [18–26]. However, the high GPU memory requirements make this approach impossible to implement for 3D images with a large number of instances. Liu et al. [27] implemented a Mask R-CNN based model for the segmentation of lung nodules on CT images. Although, lung nodules are not present in the same quantity as cysts in ADPKD, Liu et. al used a patch-based strategy to account for the dimensionality of the CT scans and limited GPU memory. In an effort to reduce computational complexity some researchers have used methods similar to the 4 color theorem for training the deep learning algorithm to predict 2D instance segmentations [39]. Although, this approach works well for 2D images, it is unclear to know how many colors would be necessary for 3D images, particularly for our problem of automatically identifying and separately distinguishing hundreds to thousands of cysts. In this study, we used a semantic instance segmentation approach, which performed very similarly to two experienced readers and, although, we used morphology operators to convert the images from instance to semantic and semantic to instance segmentations, we have shown that these operations do not have an effect on the final cyst volume and total cyst number. Future development of instance segmentation strategies may lower the need of such high memory requirements and yield the implementation of cyst instance segmentation using an object-localization/detection based method.

The main study limitation was the lack of a gold standard. The exact number of cysts and TCV values were not available; thus, we relied on the accuracy of two trained medical image readers. Another limitation was the small cohort size, which prevented us from analyzing the clinical value of these new imaging biomarkers; nonetheless, this is our main objective for future work. Lastly, the images were only from our institution acquired with the same imaging protocol. A larger dataset with samples from different institutions and machine vendors could help further generalize the cyst instance model. Additionally, this work can be extended through active learning to other MR sequences such as T2-weighted non-fat saturated images and T1-weighed images, which may add new cyst morphology information to the model. Furthermore, a similar architecture could be applied to other image modalities such as CT scans.

## Conclusions

We have developed a fully automated method for 3D instance segmentation of renal cysts in T2 MR images for ADPKD patients with mild, moderate, and severe disease. Future studies with a larger dataset are needed to better understand how these additional biomarkers relate to the patient’s disease state and prognosis.

## Supplementary Information

Below is the link to the electronic supplementary material.

Supplementary file1 (TIF 280 KB)
