# Supplementary figures and images for: Semantic Instance Segmentation of Kidney Cysts in MR Images: A Fully Automated 3D Approach Developed Through Active Learning
Source: J Digit Imaging. 2021 Apr 5;34(4):773–87. doi: 10.1007/s10278-021-00452-3 (PMC8455788; doi:10.1007/s10278-021-00452-3)

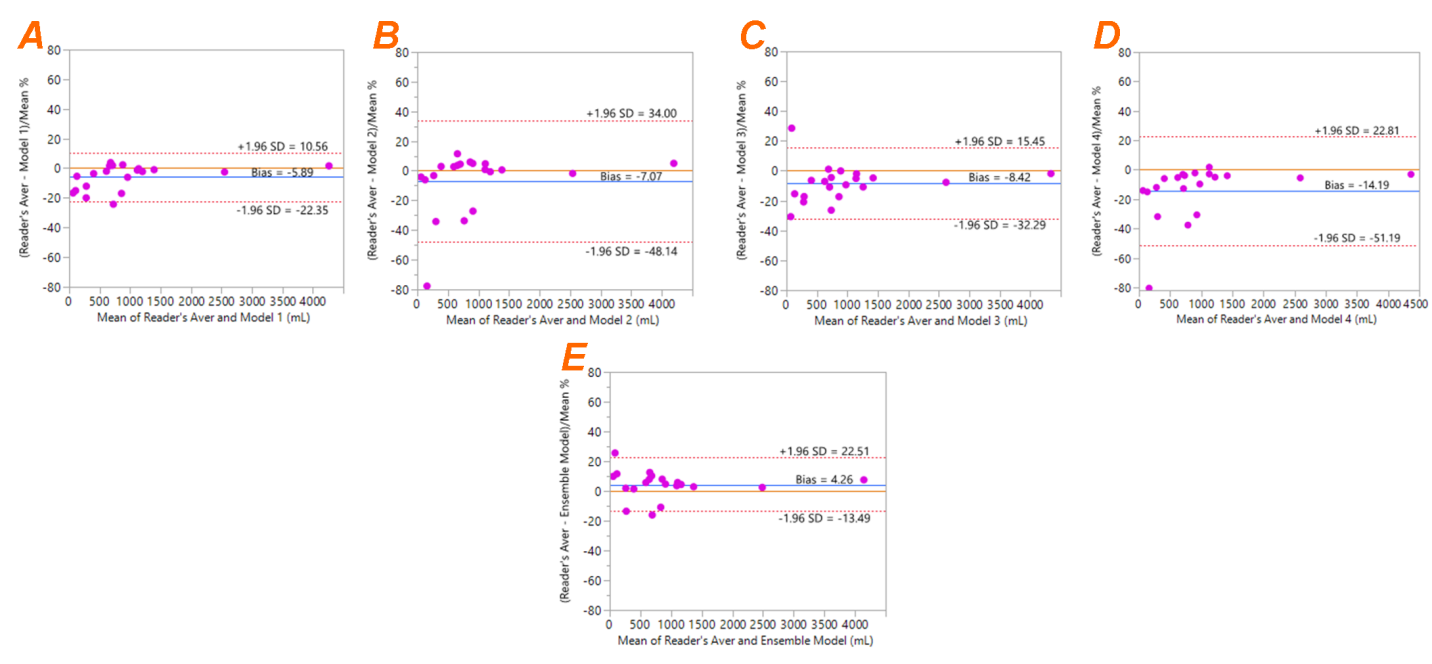

Supplement: Supplementary file 1 — Supplementary file1 (TIF 280 KB) [file 10278_2021_452_MOESM1_ESM.tif]
